# Anticytomegalovirus Peptides Point to New Insights for CMV Entry Mechanisms and the Limitations of *In Vitro* Screenings

**DOI:** 10.1128/mSphere.00586-18

**Published:** 2019-02-13

**Authors:** Joseph W. Jackson, Trevor J. Hancock, Pranay Dogra, Ravi Patel, Ravit Arav-Boger, Angela D. Williams, Stephen J. Kennel, Jonathan S. Wall, Tim E. Sparer

**Affiliations:** a Department of Microbiology, The University of Tennessee, Knoxville, Tennessee, USA; b Columbia Center for Translational Immunology, Columbia University, New York City, New York, USA; c Division of Pediatric Infectious Diseases, Johns Hopkins University School of Medicine, Baltimore, Maryland, USA; d Department of Medicine, The University of Tennessee Medical Center, Knoxville, Tennessee, USA; e Department of Radiology, The University of Tennessee Medical Center, Knoxville, Tennessee, USA; University of North Carolina, Chapel Hill

**Keywords:** HCMV, MCMV, antiviral peptides, cytomegalovirus, entry, heparan sulfate

## Abstract

Human cytomegalovirus (HCMV) is a ubiquitous betaherpesvirus that can cause severe disease following *in utero* exposure, during primary infection, or latent virus reactivation in immunocompromised populations. These complications lead to a 1- to 2-billion-dollar economic burden, making vaccine development and/or alternative treatments a high priority. Current treatments for HCMV include nucleoside analogues such as ganciclovir (GCV), foscarnet, and cidofovir. Recently, letermovir, a terminase complex inhibitor, was approved for prophylaxis after stem cell transplantation. These treatments have unwanted side effects, and HCMV is becoming resistant to them. Therefore, we sought to develop an alternative treatment that targets a different stage in viral infection. Currently, small antiviral peptides are being investigated as anti-influenza and anti-HIV treatments. We have developed heparan sulfate-binding peptides as tools for preventing CMV infections. These peptides are highly effective at stopping infection of fibroblasts with *in vitro-*derived HCMV and murine cytomegalovirus (MCMV). However, they do not prevent MCMV infection *in vivo*. Interestingly, these peptides inhibit infectivity of *in vivo-*derived CMVs, albeit not as well as tissue culture-grown CMVs. We further demonstrate that this class of heparan sulfate-binding peptides is incapable of inhibiting MCMV cell-to-cell spread, which is independent of heparan sulfate usage. These data indicate that inhibition of CMV infection can be achieved using synthetic polybasic peptides, but cell-to-cell spread and *in vivo*-grown CMVs require further investigation to design appropriate anti-CMV peptides.

**IMPORTANCE** In the absence of an effective vaccine to prevent HCMV infections, alternative interventions must be developed. Prevention of viral entry into susceptible cells is an attractive alternative strategy. Here we report that heparan sulfate-binding peptides effectively inhibit entry into fibroblasts of *in vitro-*derived CMVs and partially inhibit *in vivo-*derived CMVs. This includes the inhibition of urine-derived HCMV (uCMV), which is highly resistant to antibody neutralization. While these antiviral peptides are highly effective at inhibiting cell-free virus, they do not inhibit MCMV cell-to-cell spread. This underscores the need to understand the mechanism of cell-to-cell spread and differences between *in vivo*-derived versus *in vitro-*derived CMV entry to effectively prevent CMV’s spread.

## Abstract (precis)

In the absence of an effective vaccine to prevent HCMV infections, alternative interventions must be developed. Prevention of viral entry into susceptible cells is an attractive alternative strategy. Here we report that heparan sulfate-binding peptides effectively inhibit entry into fibroblasts of *in vitro-*derived CMVs and partially inhibit *in vivo-*derived CMVs. This includes the inhibition of urine-derived HCMV (uCMV), which is highly resistant to antibody neutralization. While these antiviral peptides are highly effective at inhibiting cell-free virus, they do not inhibit MCMV cell-to-cell spread. This underscores the need to understand the mechanism of cell-to-cell spread and differences between *in vivo*-derived versus *in vitro-*derived CMV entry to effectively prevent CMV’s spread.

## INTRODUCTION

Human cytomegalovirus (HCMV) is a significant pathogen within immunocompromised groups. Disease in these populations can result from primary infection or spontaneous latent virus reactivation (1, 2). As 60 to 90% of adults are latently infected with HCMV, there is a substantial population at risk for complications if their immune system becomes compromised (3, 4). HCMV infection/reactivation in immunocompromised persons can result in mononucleosis-like symptoms, interstitial pneumonia, gastroenteritis, retinitis, or organ transplant rejection in transplant patients (1, 3). HCMV is also the leading cause of congenital disease (5, 6). *In utero* infection may result in fetal abnormalities such as microcephaly or severe sequelae that can evolve over time in the form of progressive deafness, mental retardation, or learning disabilities (7, 8). HCMV infections impose a yearly 1- to 2-billion-dollar economic burden; therefore, development of effective treatment and preventive strategies is a high priority (5, 9). Because there is no effective vaccine, treatment of infected immunocompromised patients primarily consists of nucleoside analogs such as ganciclovir (GCV), foscarnet, or cidofovir which inhibit DNA replication (10 – 12). Unfortunately, GCV treatment can be myelosuppressive, while foscarnet and cidofovir are nephrotoxic (13). All DNA polymerase inhibitors select for resistant HCMV mutants, and cases of GCV-resistant HCMV infections are on the rise (1, 14, 15). This has led to the development of novel treatments such as the recently FDA-approved terminase inhibitor, letermovir (16).

Antiviral peptides (APs) are an attractive alternative treatment for inhibiting viral infections. Indeed, peptide therapeutics are being investigated for respiratory viruses and HIV (17 – 19). APs have different mechanisms for virus inhibition from inhibiting viral attachment, entry, replication, or egress (20). HCMV attaches to a host cell via heparan sulfate proteoglycans (HSPGs) (21). Viral glycoproteins gB and gM/gN initially interact with negatively charged sulfate moieties, which serve to “dock” the HCMV virion to the host cell (21). Docking triggers a signal cascade within the cell allowing for subsequent viral entry. HSPGs are ubiquitously expressed on most host cells, supporting the idea that HCMV can infect almost any human cell type (22).

HSPGs have a myriad of functions, including binding chemokines and cytokines and serving as scaffolds for ligand receptors, growth factors, and other cell adhesion molecules (23). Cell surface HSPGs are also major components of host-mediated endocytosis and cell membrane fusion processes. HSPG functions have been exploited for malarial and viral infections, including HCMV and herpes simplex virus 1 (24 – 26). Because of their major role in the early stages of HCMV replication, heparan sulfates (HSs) are an attractive target for intervention. HS-binding peptides effectively inhibit HCMV infection (27). However, these peptides were not tested against the more virulent *in vivo-*derived virus or in an *in vivo* setting (28).

We have previously reported that synthetic heparin-binding peptides bind pathological amyloid deposits *in vitro* and *in vivo* (29, 30). As HCMV attaches to cells via HS, we investigated whether these peptides could inhibit virus attachment. In this study, we demonstrate that these synthetic polybasic peptides are efficient at inhibiting viral entry of tissue culture-derived HCMV and murine cytomegalovirus (MCMV). We also provide evidence of effectively inhibiting an HCMV clinical isolate obtained from infected bodily secretions. However, these peptides could not prevent cell-to-cell spread of MCMV, potentially explaining the need to further investigate additional antiviral peptides for efficiency *in vivo*.

## RESULTS

### Peptide characteristics.

Three polybasic peptides, designated p5_(coil)_, p5_(coil)D_, and p5 + 14_(coil)_, were synthesized using a glycine-rich backbone to enhance flexibility of the peptide chain (Table 1**)**. The p5_(coil)_ peptide is the parental peptide (31, 32) from which the derivative p5_(coil)D_ and p5 + 14_(coil)_ peptides were designed. p5_(coil)D_ is the D form of p5_(coil)_. Because D-form peptides are more proteolytically stable and are equally effective as L-form peptides at inhibiting HCMV entry, we focused on p5_(coil)D_ for the majority of this study (33). We also utilized the peptide p5 + 14_(coil)_, which is p5_(coil)_ with an additional repeat of the last 14 amino acids. The addition of 14 amino acids has been shown to increase the efficacy of peptide-induced HCMV inhibition (27). At a peptide concentration of 50 µM, both p5_(coil)D_ and p5 + 14_(coil)_ inhibited HCMV and MCMV infection of fibroblasts (Table 1). We chose 50 µM as the initial concentration for the screening the peptides because another polybasic D-form peptide could inhibit MCMV *in vivo* at this dose (33). All three peptides were predicted to adopt a flexible coil secondary structure, which is different from previously published peptides and may increase their efficacy (34, 35).

**TABLE 1:** Polybasic peptide descriptions and characteristics^*a*^

Peptide	No. of aa	Primary structure	Property	Net charge (positive)	% of infection inhibition	
					MCMV	HCMV
p5_(coil)_	31	GGGYS KGGKG GGKGG KGGGK GGKGG GKGGK G	Flexible coil	8		
p5_(coil)D_	31	[GGGYS KGGKG GGKGG KGGGK GGKGG GKGGK G]_D_	D form of p5_(coil)_	8	68	89
p5 + 14_(coil)_	45	GGGYS KGGKG GGKGG KGGGK GGKGG GKGGK GGGKG GKGGG KGGKG	Flexible coil	12	72	96

a Peptide secondary structures were predicted via ITASSER software. Peptide inhibition of infection was determined at 50 μM. Positively charged residues are underlined.

**FIG 1:**
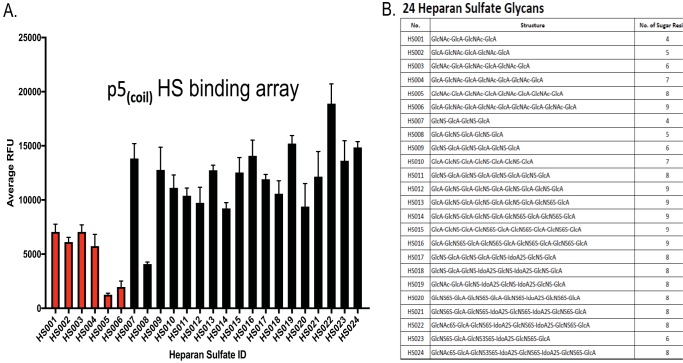
Binding of peptide p5_(coil)_ to an array of synthetic HS glycans. (A) A 0.5-mg/ml aliquot of biotinylated p5_(coil)_ peptide was incubated with a heparan sulfate glycan array, and the binding was visualized using a streptavidin-conjugated fluorophore. Nonsulfated glycans are shown in red. Each bar represents the mean and SD from 5 replicates. Statistical analysis data are presented in Table S1. (B) Composition of the HS glycans used in panel A.

As p5_(coil)_ was the peptide from which the others were generated, we tested the binding of a biotinylated variant to a panel of HS moieties using a synthetic glycoarray (Fig. 1A). p5_(coil)_ bound significantly more effectively to sulfated glycans (black bars) than to unsulfated glycans (red bars) (Fig. 1A), with the exception of the 5-sugar HS008 glycan. Statistical analysis showed significantly enhanced binding of p5_(coil)_ to almost all sulfated glycans relative to nonsulfated species (see Table S1 in the supplemental material). In general, no significant difference was observed between levels of peptide binding to the structurally different sulfated HSs. Figure 1B lists the structure of the glycans used in the glycan array. These results highlight that p5_(coil)_ preferentially binds sulfated glycans.

10.1128/mSphere.00586-18.1 TABLE S1 Statistical analysis of the glycan array. Statistical significance was determined by an ordinary one-way ANOVA with Tukey’s multiple comparison of means. ns, not significant; *, *P* ≤ 0.05; **, *P* ≤ 0.01; ***, *P* ≤ 0.001; ****, *P* ≤ 0.0001. Analysis was carried out using GraphPad Prism. Download Table S1, DOCX file, 0.2 MB. Copyright © 2019 Jackson et al. 2019 Jackson et al. This content is distributed under the terms of the Creative Commons Attribution 4.0 International license.

### Efficacy of inhibition of CMV infection.

**FIG 2:**
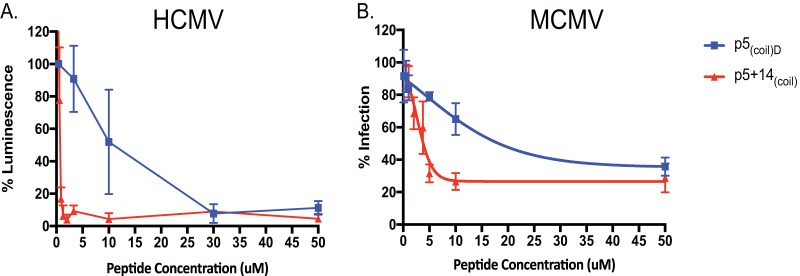
The p5_(coil)_ family of peptides prevent HCMV and MCMV infection. Peptides were serially diluted and assayed in an HCMV (TB40/E UL18Luc) luciferase assay (A) or an MCMV plaque reduction assay (B). IC_50_s for HCMV of 9.98 μM for p5_(coil)D_ and 0.6 μM for p5 + 14_(coil_**_)_** and IC_50_s for MCMV of 22.6 μM for p5_(coil)D_ and 2.97 μM for p5 + 14**_(_**_coil_**_)_** were calculated with GraphPad Prism. Each point represents an average of 3 or 4 replicates ± SD from 3 experiments.

**FIG 3:**
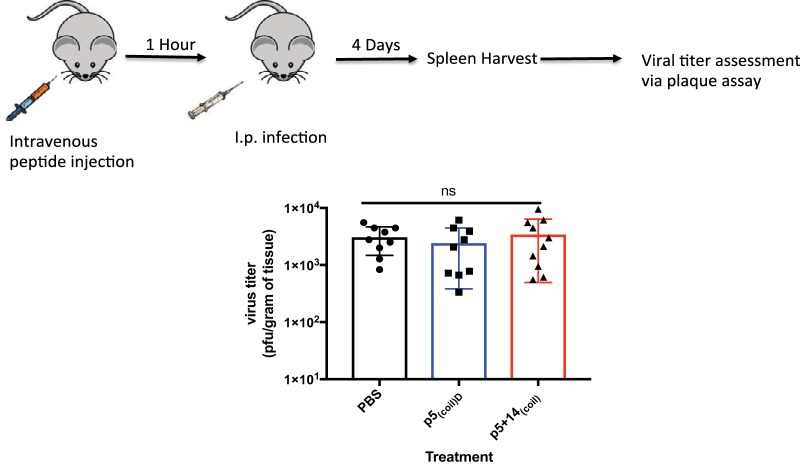
Peptide efficacy *in vivo*. BALB/c mice were treated with peptide (250 μg/mouse) i.v. and infected with 1 × 10^6^ PFU of MCMV i.p. 1 h later. Bars represent the average of 4 or 5 mice per group from 2 experiments. One-way ANOVA with Tukey’s multiple comparison of means was used to determine statistical significance. ns, not significant.

The blockade of HCMV and MCMV attachment to cells was studied in the presence of increasing concentrations of peptide p5_(coil)D_ or p5 + 14_(coil)_ (Fig. 2). The estimated 50% inhibitory concentrations (IC_50_s) of p5_(coil)D_ and p5 + 14_(coil)_ for blocking HCMV (TB40/E) were 9.98 and 0.6 μM, respectively (Fig. 2A), and those for MCMV were 22.6 and 2.97 μM, respectively (Fig. 2B). These data indicate that our peptides are capable of inhibiting both HCMV and MCMV; however, MCMV is inhibited to a lesser extent. Peptide inhibition of infection of mice was evaluated using the MCMV mouse model (Fig. 3). BALB/c mice were pretreated with p5_(coil)D_ or p5 + 14_(coil)_ at 250 μg per mouse 1 h prior to infection. Evaluation of the viral titer in spleens harvested 4 days postinfection (dpi) indicated no significant difference in viral burden (Fig. 3). In order to confirm peptide was present at the time of infection and following infection, we evaluated peptide biodistribution postadministration (see Table S2 in the supplemental material). Our biodistribution assay confirms that p5_(coil)D_ or p5 + 14_(coil)_ is present within the host at the time of infection and following infection. While there is a difference in biodistributions between the two peptides, there is no difference in levels of viral dissemination to the spleen. We have previously reported the inability of p5R_D_, another antiviral polybasic peptide, which is significantly more stable *in vivo*, to substantially inhibit infection of any primary dissemination organs (e.g., spleen, liver, and lung) (33). The inability of these peptides to reduce viral load *in vivo* could be due *in vivo* dosage/timing effect, but an alternative explanation is that the peptides differ in their ability to block *in vivo*-derived virus versus *in vitro*-derived viruses. Previous studies have reported differences between MCMVs grown in culture compared with those harvested *in vivo*, which was related to their HSPG usage for entry (36 – 40).

10.1128/mSphere.00586-18.2 TABLE S2 p5_(coil)D_ or p5 + 14_(coil)_ biodistribution in BALB/c mice. Mice were injected with 250 μg/mouse of ^125^I-labeled peptide. Mice were euthanized at 1 and 4 h postinjection (hpi), and the indicated organs were harvested. The amount of ^125^I present in each organ was measured. Data represent the percentage of injected dose (ID)/g of tissue. Download Table S2, DOCX file, 0.3 MB. Copyright © 2019 Jackson et al. 2019 Jackson et al. This content is distributed under the terms of the Creative Commons Attribution 4.0 International license.

### Peptide inhibition of *in vivo*- and *in vitro*-derived MCMV.

**FIG 4:**
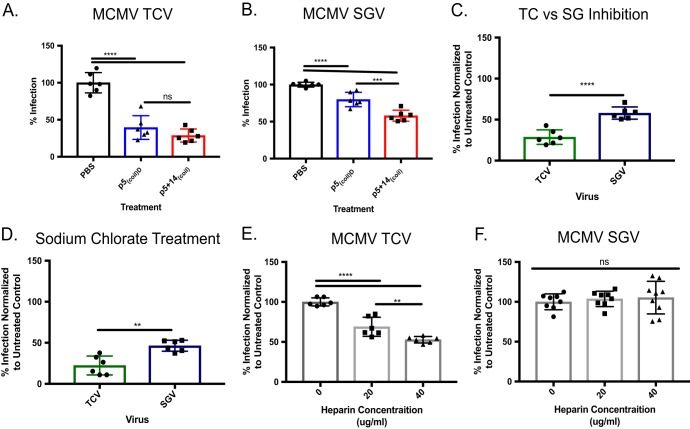
Differential effects of peptide treatment on *in vivo*- and *in vitro*-derived MCMV. Peptide at a 50 µM concentration was used to inhibit (A) tissue culture-derived (TCV) or (B) salivary gland-derived (SGV) MCMV. (C) Data from panels A and B to compare the efficacy of p5 + 14_(coil)_ inhibition of TCV and SGV. (D) MEF 10.1 cells were treated with 50 mM sodium chlorate to remove 2-O- and 6-O-linked sulfations. Treated cells were infected with ∼100 PFU of TCV or SGV. Either TCV (E) or SGV (F) was incubated with various concentrations of heparin and used to infect MEF 10.1 cells (∼100 PFU/well). Data were normalized to untreated (PBS) controls. Bars represent the average ± SD from 2 experiments with 3 replicates per experiment. Statistical significance was determined by one-way ANOVA with a Tukey’s multiple comparison of means. ns, not significant; **, *P* ≤ 0.01; ***, *P* ≤ 0.001; ****, *P* ≤ 0.0001.

To evaluate the possibility of differential inhibition between *in vitro-*derived virus and *in vivo-*derived virus, we performed plaque reduction assays using MCMV salivary gland-isolated virus (SGV) and MCMV passaged on cultured cells (tissue culture-derived virus [TCV]) (Fig. 4). Both p5_(coil)D_ and p5 + 14_(coil)_ significantly inhibited infection of both murine TCV (Fig. 4A) and SGV (Fig. 4B) relative to untreated cells (phosphate-buffered saline [PBS]). Additionally, in both cases p5 + 14_(coil)_ was significantly more efficacious than p5_(coil)D_. When used at 50 μM, p5 + 14_(coil)_ inhibited murine TCV (75%) more effectively than SGV (40%) (Fig. 4C). Our data support observations made by Ravindranath and Graves (36) and indicate SGV and TCV use different entry strategies. One entry mechanism is inhibited by peptides (i.e., TCV), and the other is only partially blocked (i.e., SGV).

To further investigate the differences in SGV and TCV entry identified by the peptide inhibition studies, mouse embryonic fibroblasts (MEFs) were treated with 50 mM sodium chlorate prior to infection to remove 2-O- and 6-O-linked HS sulfations (41). We focused on these sulfation patterns based on observations from HCMV, which indicated that these O-linked sulfations were important for viral attachment (28). This treatment resulted in inhibition of infection of both SGV and TCV, with the latter being significantly more impacted (Fig. 4D). It is known that incubation of MCMV with heparin blocks cellular entry; therefore, we studied the effect of increasing heparin concentration on infection efficiency of TCV (Fig. 4E) and SGV (Fig. 4F). Pretreatment of TCV with heparin resulted in a dose-dependent decrease in infection, with 50% loss of efficiency in the presence of 40 µg/ml heparin (Fig. 4E). In contrast, there was no significant decrease in the infectivity of murine SGV following pretreatment with 40 µg/ml heparin (Fig. 4F).

**FIG 5:**
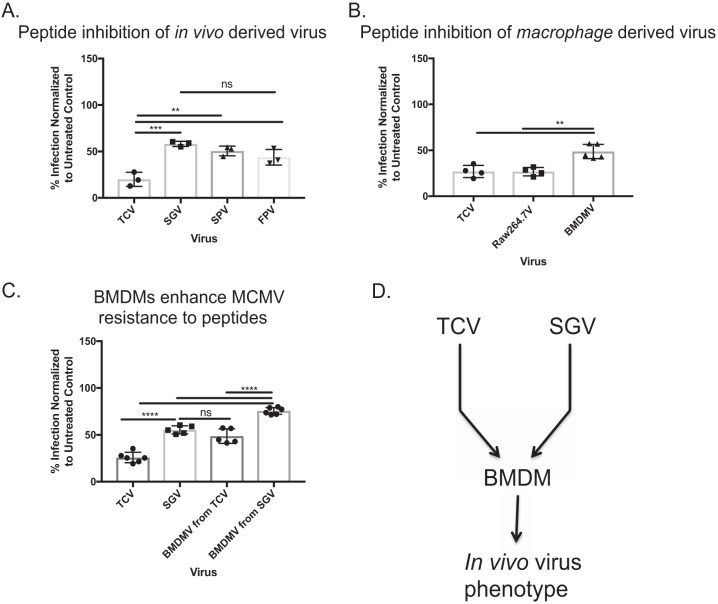
Generation of peptide-resistant MCMV *in vitro.* (A) Virus was harvested from salivary gland (SGV), spleen (SPV), and footpads (FPV) of mice infected with MCMV. MEF 10.1 cells were treated with 50 µM p5 + 14_(coil)_ and then infected with ∼100 PFU of each virus. The percentage of infection inhibition was determined by comparison to untreated controls. (B) TCV was grown on RAW 264.7 macrophages and BMDMs. Progeny virus was then subjected to a plaque reduction assay. The percentage of virus inhibition was determined by comparison to untreated controls. (C) Comparison of peptide inhibition of MEF 10.1 cells infected with TCV, SGV, or BMDM-derived virus (BMDMV) originally from TCV or SGV. Bars represent the average ± SD from 2 experiments with 2 or 3 replicates per experiment. Statistical significance was determined by one-way ANOVA with Tukey’s multiple comparison of means with a two-tailed *t* test. ns, not significant; *, *P* ≤ 0.05; **, *P* ≤ 0.01; ***, *P* ≤ 0.001; ****, *P* ≤ 0.0001. (D) Schematic of method used to generate *in vivo-*like virus *in vitro*.

Because viruses derived from different tissues vary in their susceptibility to antibody neutralization (37), we speculated that perhaps not all *in vivo-*derived MCMVs would be resistant to peptide inhibition. Therefore, we infected mice subcutaneously in the footpad, and SGV was isolated on day 14 postinfection, splenic virus (SPV) at day 5, and the footpad virus (FPV) at day 3. The different time points were necessary to maximize viral load in the given organ. Once MCMV organ titers were determined, whole-organ homogenates were plated at ∼100 PFU on peptide-treated MEF 10.1 cells (Fig. 5A). As expected there was some inhibition of *in vivo*-grown virus, but not to the same levels as TCV. Interestingly, there was no difference between the different organs in regard to peptide inhibition susceptibility. Because there is no difference between viruses isolated from different organs, the SGV phenotype is not the result of SGV sample preparation.

We speculated that the differences in susceptibility to the peptides correlated with differences in entry complexes that determine cellular tropism (42). MCMVs grown on macrophages contain different entry complexes compared to MCMVs grown on fibroblasts (42). Therefore, we tested whether MCMVs derived from macrophages *in vitro* would mimic peptide inhibition of SGV or TCV. *In vitro-*derived MCMV was grown on the macrophage cell line RAW 264.7 or bone marrow-derived macrophages (BMDMs). The progeny viruses were subjected to a plaque reduction assay (Fig. 5B). Peptide treatment of cells blocked RAW 264.7-grown virus (∼75% inhibition) but only partially blocked BMDM virus (∼50% inhibition). Not only are BMDMs capable of generating “*in vivo-*like” virus, but when SGV virus was grown on BMDMs, the peptide was even less efficient in blocking MCMV infection (Fig. 5C). Regardless of the initial MCMV input into BMDMs, they produced “*in vivo-*like” virus (Fig. 5B to D). Additionally, these results indicate that the phenotype observed with SGV and virus isolated from other tissues is not an artifact of sample preparation.

### Peptide inhibition of *in vivo*-derived HCMV.

**FIG 6:**
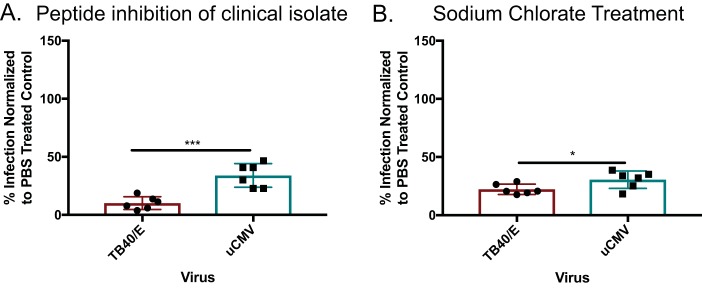
Peptide and sodium chlorate treatment prevents urine-derived CMV (uCMV) infection. (A) MCR-5 human fibroblasts were treated with p5 + 14**_(_**_coil)_ at 50 µM and infected with the TB40/E strain or uCMV. (B) MRC-5 cells were treated with 50 mM sodium chlorate to remove 2-O- and 6-O-linked sulfations. All cells were infected with ∼100 PFU of TB40/E or clinically derived HCMV (uCMV). The percentage of infection was normalized to PBS-treated controls. Bars are the averages of replicates from two experiments ± SD. Statistical significance was determined by unpaired *t* test. *, *P* ≤ 0.05; ***, *P* ≤ 0.001.

We sought to determine whether p5 + 14_(coil)_ could inhibit infection of an HCMV clinical isolate. This virus was used directly from a patient’s urine (11). This is important because the tropism/entry complex can mutate after a single passage *in vitro* (43, 44). Peptide p5 + 14_(coil)_ significantly inhibited the entry of a clinical isolates of HCMV into human fibroblasts (∼70% inhibition [Fig. 6A]), but was significantly more effective when tissue culture-grownTB40/E virus was used (∼90% inhibition). Because these results mimicked MCMV’s peptide inhibition, we tested which HS moieties were important for viral entry. MRC-5 fibroblasts were treated with 50 mM sodium chlorate to remove 2-O and 6-O sulfations (Fig. 6B). Despite the small but significant difference between HCMVs from *in vitro-* or *in vivo-*grown viruses, removing the 2-O and 6-O sulfations prevented infection of both HCMVs, albeit more inhibition of tissue culture-grown HCMVs than the clinical isolate was observed. These results corroborate the MCMV data. Both viruses highly rely on sulfated surface glycans for entry.

### Do polybasic peptides prevent MCMV cell-to-cell spread?

**FIG 7:**
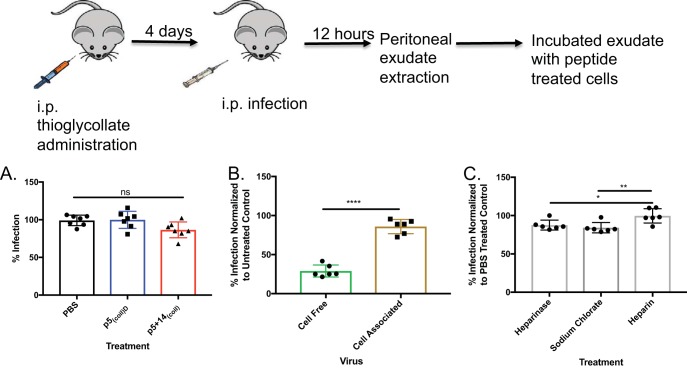
Peptide inhibition of MCMV cell-to-cell spread. (A) MEF 10.1 cells were treated with PBS, p5_(coil)_, or p5 + 14_(coil)_ at 100 μM 1 h prior to incubation with 1 × 10^5^ MCMV-infected peritoneal exudate cells. (B) MEF10.1 cells were treated with p5 + 14_(coil)_ at 50 μM 1 h prior to incubation with either cell-free MCMV or 300 MEF 10.1 cells infected with MCMV. (C) MEF 10.1 cells were treated with 12 U/μl of heparinase for 1 h, 50 mM sodium chlorate overnight, or 40 µg/ml of heparin and then infected with cell-associated MCMV. Bars represent the average from 2 experiments with 3 or 4 replicates per experiment. Statistical significance was determined by an ordinary one-way ANOVA with a Tukey’s multiple comparison of means or an unpaired *t* test. ns, not significant; *, *P* ≤ 0.05; **, *P* ≤ 0.01; ****, *P* ≤ 0.0001.

Cell-to-cell spread *in vivo* is important for viral dissemination within the host (11, 45, 46). We evaluated the effectiveness of p5_(coil)D_ and p5 + 14_(coil)_ on cell-to-cell spread. Infected peritoneal exudate cells (PECs) were harvested from MCMV-infected mice and coincubated with MEF 10.1 cells, treated with 100 μM p5_(coil)D_, p5 + 14_(coil)_, or PBS as a control. Inhibition of MCMV infection was measured via plaque reduction assay. Peptide treatment has no effect on cell-to-cell spread (Fig. 7A). Because most of the PEC population consists of immune cells (data not shown), we tested our peptide’s ability to inhibit cell-to-cell spread from infected to uninfected MEF 10.1 cells in a plaque reduction assay. As seen with the PECs, peptide treatment did not inhibit cell-to-cell spread regardless of cell type (Fig. 7B). To evaluate whether or not HS mediates cell-to-cell spread, we treated murine fibroblasts with heparinase, sodium chlorate, and heparin. Cell-associated virus spread was marginally affected in the absence of HS, pointing to a different entry mechanism from cell-free MCMV, as heparin treatment was ineffective at inhibiting infection (Fig. 7C). These data highlight the complexity of CMV entry whether the virus enters from cell-to-cell spread or cell-free virus.

## DISCUSSION

Decades of investment and innovation have failed to generate a protective HCMV vaccine (10, 11). Without a vaccine, treatment of HCMV relies on drugs that target two different stages of HCMV replication. First, nucleoside analogues (e.g., GCV, foscarnet, and cidofovir) have been the standard treatment for HCMV disease, but these compounds are myelosuppressive and nephrotoxic. The recently approved letermovir (trade name Prevymis), which inhibits the terminase complex, is not myelosuppressive or nephrotoxic (16). All of these agents potentially select for resistant viruses (15, 47). Therefore, a multifaceted approach may be required to inhibit additional stages during HCMV replication, and this could include APs that block viral entry. Other anti-CMV APs inhibit viral entry of *in vitro-*derived CMV, but none have been shown to inhibit a clinical isolate of HCMV (27, 28, 33, 48). We demonstrated that our p5_(coil)_ peptides are capable of inhibiting infection of both HCMV and MCMV and that these peptides can partially inhibit infection of *in vivo-*derived CMVs. This is the first report of an effective inhibition of HCMV isolated directly from urine. Clinical isolates have previously been shown to be resistant to anti-HCMV antibody neutralization (49). Although inhibition of *in vivo-*derived virus was not as efficient as the blockade of tissue culture*-*derived virus, greater than 50% reduction in infectivity was achieved, which is comparable to that observed following sodium chlorate treatment of cells. These results support previous observations and point to the important role of 2-O- or 6-O-sulfated cell surface glycans in viral entry via HS moieties (28).

Our data indicate that the p5_(coil)_ peptides, which presumably inhibit the interactions of viral attachment proteins with HS, were unable to inhibit cell-to-cell spread. Also, heparin treatment of infected cells did not inhibit cell-to-cell spread. These data provide insights into why polyclonal antibodies generated in response to the gB vaccine are of limited efficacy (50) (i.e., the mechanism of HCMV cell-to-cell spread may be independent of HS interactions involving the viral gB protein). The studies of peptide-mediated inhibition of viral attachment and infection have elucidated several important aspects of CMV infection pathways. First, these peptides, which bind preferentially to sulfated HS, effectively inhibited TCV entry into cultured cells. There was one HS exception, HS008, to which the peptide did not bind. This glycan consisted of GlcA-GlcNS-ClcA-GlcNS-GlcA, while HS007 is one sugar moiety shorter but binds to the peptide very well. The full set of features that are important for viral entry, (i.e., length, number of repeating sugar units, etc.) and whether it depends on *in vivo*- or cell culture-grown virus remain to be determined. Interestingly, another AP, p5 + 14, also did not bind to HS008 (data not shown) (27). Could the lack of binding to HS008 be the reason that the peptides cannot block 100% of the infection? Without knowing the composition of the HS on the cell surface, we can only speculate the lack of complete inhibition of infectivity (i.e., 5% remaining infectivity for HCMV and 30% for MCMV). Entry differences could be due to differences in entry complexes, glycosylations of the virion proteins, or other factors during replication *in vivo*. Because we have demonstrated that virus grown on BMDMs recapitulates the “*in vivo*” virus phenotype, this will allow an in-depth investigation of these possibilities. Our data demonstrate the significant biochemical differences between *in vivo*-grown virus and virus cultured *in vitro*. Previously, differences between *in vivo*-derived and tissue culture-grown MCMVs were defined as “virulent” for *in vivo*-grown virus or “attenuated” when grown *in vitro*. These differences were not due to selection of mutated viruses, but rather differential usage of sugar moieties for entry (36, 39, 40), which could explain why one may be more virulent than the other. Interestingly, it appears that all *in vivo*-derived viruses, regardless of the tissue from which they were isolated, utilize discrete surface HS compared to tissue culture-derived virus. These differences in HS usage may explain the lack of peptide-mediated inhibition of *in vivo*-grown MCMV. Furthermore, it is noteworthy that cell attachment by *in vivo-*grown virus did not depend on 2-O or 6-O glycans, as indicated by the differential inhibition following sodium chlorate treatment of the cells (Fig. 6).

Perhaps most notably, we have shown that the HS-binding peptides do not inhibit cell-to-cell spread of CMV, which represents the main mechanism of cellular transmission *in vivo* (45, 51 – 53). Our data indicate that cell-to-cell spread does not require HS (i.e., heparinase and sodium chlorate treatment of uninfected cells prior to being cocultured with infected fibroblasts only achieved approximately 15% inhibition). These data once again indicate that our current understanding of CMV entry remains incomplete. Further understanding of the mechanism of cell-to-cell spread may provide a path to improve treatment strategies for HCMV.

## MATERIALS AND METHODS

### Cells and viruses.

All experiments were performed with low-passage cells (<20 passages). MRC-5 human lung fibroblasts were cultured in modified Eagle’s medium (MEM; Lonza, Rockland, ME) supplemented with 10% fetal bovine serum (FBS; Atlanta Biologicals, Flowery Branch, GA), 1% penicillin–streptomycin, and 1% l-glutamine. Cells of the mouse embryonic fibroblast (MEF) line 10.1 (54) were cultured in Dulbecco’s modified Eagle’s medium (DMEM; Lonza, Rockland, ME) supplemented with 10% Fetalclone III serum (HyClone, Logan, UT), 1% penicillin–streptomycin, and 1% l-glutamine. RAW 264.7 cells were cultured in DMEM (Lonza, Rockland, ME) with 10% Fetalclone III (HyClone, Logan, UT), 1% penicillin–streptomycin, and 1% l-glutamine. Primary bone marrow-derived macrophages (BMDMs) were cultured in RPMI 1640 (Lonza, Rockland, ME) supplemented with 10% Fetalclone III (HyClone, Logan, UT), 1% penicillin–streptomycin, and 1% l-glutamine.

The HCMV TB40/E and MCMV K181 (55) were used in this study. HCMV TB40/E expressing luciferase under the control of the UL18 promoter was a gift from Christine O’Connor and Eain Murphy (University of Buffalo and Forge Life Science, LLC). TB40/E viruses were propagated on HUVECs. MCMV was produced *in vitro* using MEF 10.1 cells. All viruses were stored at −80°C until use. Viral titer was assessed by plaque assay (described below) on MEF 10.1 cells (MCMV) or MRC-5 cells (HCMV). The HCMV clinical isolates were collected at Johns Hopkins University without any identifiers that can link them to a specific patient. To generate BMDM virus, differentiated BMDMs were infected with K181 and virus was harvested after a 100% cytopathic effect (CPE) was observed. Salivary gland-derived virus was harvested from mouse salivary glands at 14 dpi with K181 via the intraperitoneal (i.p.) route. Spleen-derived virus was obtained by harvesting the organ 5 dpi after i.p. infection, while footpad-derived virus was obtained by harvesting footpads 3 dpi following footpad inoculation with K181. All organs were homogenized, and titers were determined via plaque assay.

### BMDM differentiation.

Bone marrow from naive mice was harvested and incubated in RPMI 1640 for 4 h. The medium was then changed and supplemented with macrophage colony-stimulating factor (M-CSF; PeproTech, Rocky Hill, NJ) at 1 ng/μl for 7 days. The medium was changed every 3 days. Differentiated cells were infected with K181 at a multiplicity of infection (MOI) of ∼0.1. Virus was harvested 1 day after cells showed 100% CPE.

### Treatment of cells and viruses.

Cells were washed with PBS prior to addition of treatment. Heparinase I (NEB, Ipswich, MA) was used at 12 U/μl in medium. Cells were pretreated for 1 h at 37°C before addition of virus or cells. MEF 10.1 or MRC-5 cells were treated for 1 h with 50 mM sodium chlorate to remove 2-O- and 6-O-linked HS as previously described (41). Heparin at various concentrations was preincubated with virus or infected fibroblasts for 30 min at 4°C. Following pretreatment, viral infectivity was assayed by plaque assay or cell-to-cell transfer assay as described below.

### Peptides.

Peptides were purchased and purified as previously described (27). Briefly, peptides were purified by high-performance liquid chromatography (HPLC), and purity was confirmed by mass spectrometry (MS). Purified peptides were lyophilized and resuspended in PBS prior to use.

### Plaque reduction assay.

Lyophilized peptides were resuspended in PBS and stored at 4°C until use. Fibroblasts were seeded into 24-well dishes. After cells reached ∼80% confluence, medium was removed and cells were washed with PBS. Peptide in PBS plus 10% FBS or PBS plus 10% FBS alone as the control was added, and the mixture was incubated for 30 min at 37°C. Virus was added to treated and untreated control wells (∼100 PFU/well) and incubated for an additional hour. Following virus incubation, medium was removed and the overlay was added. Overlays consisted of 0.75% carboxymethyl cellulose (CMC; Sigma-Aldrich, St. Louis, MO) for MCMV-infected wells and 0.5% SeaKem agarose (Lonza, Rockland, ME) in complete medium for HCMV-infected wells. For MCMV assays, plates were incubated for 5 days, at which point they were stained with Coomassie stain and plaques counted using a dissecting microscope. Reduction in viral infectivity was expressed as a percentage of infectivity of PBS-treated wells. Data were analyzed using Prism 7 (GraphPad Software, La Jolla, CA).

### Cell-to-cell transfer of virus.

To examine fibroblast-mediated cell-to-cell spread, MEF 10.1 cells were infected with K181 at an MOI of 3. Following 16 h of incubation, cells were trypsinized, washed, and counted. Approximately 300 potentially infected cells were added to an uninfected 100% confluent monolayer of MEF 10.1 cells. The uninfected monolayer was pretreated with peptide or PBS. Infected cells were given an hour to transfer infection, medium was removed, and a CMC overlay was added. In order to evaluate immune cell-mediated cell-to-cell spread, mice were injected i.p. with 3% thioglycollate (56). After 4 days, animals were infected i.p. with MCMV K181 at 1 × 10^6^ PFU, and peritoneal exudate cells (PECs) were collected 12 h postinfection. PECs were added to an uninfected monolayer either treated with p5 + 14_(coil)_ or untreated. PECs were incubated for an hour, medium was removed, and a CMC overlay was added. Analysis was performed as described for the plaque reduction assay.

### HCMV infectivity assay.

HCMV TB40/E infectivity was assessed using a luciferase reporter assay as previously described (33). Briefly, MRC-5 fibroblasts were seeded into a 24-well dish. After reaching ∼80% confluence, cells were washed once with PBS and peptide with PBS plus 10% FBS or the PBS-plus-10% FBS control was incubated at 37°C for 30 min. Virus was added at ∼1,000 relative light units (RLU) and incubated at 37°C for 1 h. Cells were washed, medium was replaced, and then the mixture was incubated at 37°C for 3 days. On day 3, cells were washed with PBS and lysed using passive lysis buffer (Promega, Madison, WI), and the cell lysates were pelleted. Luciferase reagent (Gaussian luciferase) was combined 1:1 with cell lysate in a clear-bottom 96-well plate. Luminescence was measured using a Synergy 2 plate reader (BioTek, Winooski, VT) and recorded as RLU. The RLU from untreated uninfected samples was subtracted as background, and results were normalized to untreated infected wells to 100% infection. Data were analyzed using Prism 7 (GraphPad Software, La Jolla, CA). The data were expressed as the percentage of luminescence of the untreated infected well.

### Statistical analysis and IC_50_ determination.

Each experiment represents two or more independent experiments with at least three replicates per experimental group, unless otherwise indicated. Individual data points are shown for all graphs. Error bars represent the standard deviation (SD) from each data set. Statistical significance was determined by one-tailed Student's *t* test or one-way analysis of variance (ANOVA) with Tukey’s multivariance analysis when appropriate. IC_50_ values were calculated using a linear regression sigmoidal dose-dependent test. All statistical analysis was performed using Prism 7 (GraphPad Software, La Jolla, CA). Statistical significance was assigned to *P* values of <0.05. Significant values are labeled as follows: ns, not significant; *, *P* ≤ 0.05; **, *P* ≤ 0.01; ***, *P* ≤ 0.001; and ****, *P* ≤ 0.0001.

### Glycan array.

The glycan array was performed by Z-biotech, LLC. Briefly, biotinylated peptide was incubated with various heparan sulfate derivatives. Following incubation, an antibiotin, fluorescently labeled antibody was added, and a plate reader determined fluorescence. The data represent 6 technical replicates per HS residue.

### Mice.

Animal use was approved under The University of Tennessee, Knoxville, IACUC protocol. The animals used were housed and bred at the Walters Life Science Laboratory Animal Facility at The University of Tennessee. BALB/c mice (6 to 12 weeks) were purchased from Jackson Laboratory (Bar Harbor, ME) and housed under specific-pathogen-free conditions.

### *In vivo* analysis of peptide efficacy.

Mice were treated with intravenously via the retro-orbital route with the indicated peptide at 250 μg/mouse 1 h prior to infection. One hour posttreatment, animals were infected i.p. with 1 × 10^6^ PFU of K181. Four days postinfection, animals were euthanized and spleens were harvested. Viral burden was determined by plaque assay as described above.

### Peptide biodistribution.

To determine the distribution of our APs *in vivo*, mice were injected intravenously (i.v.) in the lateral tail vein with ^125^I-labeled peptides (<120 μCi, 20 μg of peptide). At 1 and 4 h postinjection, mice were euthanized with an isoflurane inhalation overdose, spleen, liver, lung, and nine other tissues were harvested, and the tissue radioactivity was measured as previously described (30, 33). The biodistribution of radiolabeled peptide was expressed as a percentage of the injected dose per gram of tissue.
