# Supplementary material for: Anticytomegalovirus Peptides Point to New Insights for CMV Entry Mechanisms and the Limitations of In Vitro Screenings
Source: mSphere. 2019 Feb 13;4(1):e00586-18. doi: 10.1128/mSphere.00586-18 (PMC6374593; doi:10.1128/mSphere.00586-18)
Supplement: Supplementary file 1 [file mSphere.00586-18-st001.docx]

	HS001	HS002	HS003	HS004	HS005	HS006	HS007	HS008	HS009	HS010	HS011	HS012	HS013	HS014	HS015	HS016	HS017	HS018	HS019	HS020	HS021	HS022	HS023	HS024
HS001		ns	ns	ns	****	****	****	ns	****	****	*	ns	****	ns	****	****	****	***	****	ns	****	****	****	****
HS002	ns		ns	ns	****	***	****	ns	****	****	***	***	****	**	****	****	****	****	****	**	****	****	****	****
HS003	ns	ns		ns	****	****	****	ns	****	***	*	ns	****	ns	****	****	****	**	****	ns	****	****	****	****
HS004	ns	ns	ns		****	**	****	ns	****	****	****	***	****	**	****	****	****	****	****	**	****	****	****	****
HS005	****	****	****	****		ns	****	ns	****	****	****	****	****	****	****	****	****	****	****	****	****	****	****	****
HS006	****	***	****	**	ns		****	ns	****	****	****	****	****	****	****	****	****	****	****	****	****	****	****	****
HS007	****	****	****	****	****	****		****	ns	ns	*	****	ns	****	ns	ns	ns	**	ns	****	ns	****	ns	ns
HS008	ns	ns	ns	ns	ns	ns	****		****	****	****	****	****	****	****	****	****	****	****	****	****	****	****	****
HS009	****	****	****	****	****	****	ns	****		ns	ns	*	ns	***	ns	ns	ns	ns	ns	**	ns	****	ns	ns
HS010	****	****	***	****	****	****	ns	****	ns		ns	ns	ns	ns	ns	*	ns	ns	****	ns	ns	****	ns	***
HS011	*	***	*	****	****	****	*	****	ns	ns		ns	ns	ns	ns	**	ns	ns	****	ns	ns	****	*	****
HS012	ns	***	ns	***	****	****	****	****	*	ns	ns		*	ns	*	****	ns	ns	****	ns	ns	****	***	****
HS013	****	****	****	****	****	****	ns	****	ns	ns	ns	*		***	ns	ns	ns	ns	ns	**	ns	****	ns	ns
HS014	ns	**	ns	**	****	****	****	****	***	ns	ns	ns	***		**	****	ns	ns	****	ns	*	****	****	****
HS015	****	****	****	****	****	****	ns	****	ns	ns	ns	*	ns	**		ns	ns	ns	ns	**	ns	****	ns	ns
HS016	****	****	****	****	****	****	ns	****	ns	*	**	****	ns	****	ns		ns	**	ns	****	ns	****	ns	ns
HS017	****	****	****	****	****	****	ns	****	ns	ns	ns	ns	ns	ns	ns	ns		ns	**	ns	ns	****	ns	*
HS018	***	****	**	****	****	****	**	****	ns	ns	ns	ns	ns	ns	ns	**	ns		****	ns	ns	****	*	****
HS019	****	****	****	****	****	****	ns	****	ns	****	****	****	ns	****	ns	ns	**	****		****	*	**	ns	ns
HS020	ns	**	ns	**	****	****	****	****	**	ns	ns	ns	**	ns	**	****	ns	ns	****		*	****	****	****
HS021	****	****	****	****	****	****	ns	****	ns	ns	ns	ns	ns	*	ns	ns	ns	ns	*	*		****	ns	ns
HS022	****	****	****	****	****	****	****	****	****	****	****	****	****	****	****	****	****	****	**	****	****		***	***
HS023	****	****	****	****	****	****	ns	****	ns	ns	*	***	ns	****	ns	ns	ns	*	ns	****	ns	***		ns
HS024	****	****	****	****	****	****	ns	****	ns	***	****	****	ns	****	ns	ns	*	****	ns	****	ns	***	ns	

Key:	ns	*	**	***	****	Unsulfated HS
