# Supplementary material for: Anticytomegalovirus Peptides Point to New Insights for CMV Entry Mechanisms and the Limitations of In Vitro Screenings
Source: mSphere. 2019 Feb 13;4(1):e00586-18. doi: 10.1128/mSphere.00586-18 (PMC6374593; doi:10.1128/mSphere.00586-18)
Supplement: Supplementary file 2 [file mSphere.00586-18-st002.docx]

Tissue Distribution	$p5_{(coil)D}$				$p5{+}14_{(coil)}$			
%ID/gram tissue	1hpi Average	SD	4hpi Average	SD	1hpi Average	SD	4hpi Average	SD
Muscle	0.42	0.02	0.13	0.04	1.3	0.21	0.17	0.01
Liver	3.27	0.41	3.17	0.47	7.23	1.06	0.82	0.07
Pancreas	0.67	0.11	0.2	0.05	2.79	0.17	0.44	0.04
Spleen	1.21	0.24	0.64	0.08	3.72	0.31	0.65	0.1
Left kidney	189.23	42.96	189.75	48.81	16.25	0.72	1.93	0.62
Right kidney	161.54	33.66	234.86	53.72	16.58	1.05	1.93	0.09
Stomach	3.36	1.25	1.01	0.28	14.4	1.26	3.08	0.7
Upper intestine	1.15	0.18	0.44	0.16	3.91	0.43	0.88	0.06
Lower intestine	1.18	0.08	0.37	0.04	3.53	0.33	0.58	0.03
Heart	0.69	0.08	0.24	0.11	2.03	0.38	0.29	0.01
Lung	2.08	0.17	0.94	0.29	5.15	0.27	0.9	0.13
Blood	1.3	0.07	0.22	0.09	3.53	0.18	0.53	0.05
